# Surgical Hints

**Published:** 1899-05

**Authors:** 


ARTICEL XII.

### SURGICAL HINTS.

Do not cough or sneeze over the operative field.

Children are more easily affected than adults by carbolic acid. Avoid its use in the young, as a number of cases of hematuria have resulted in them from the employment of wet dressings.

When strapping a chest for fractured ribe, it sometimes occurs that, instead of affording relief, the pain is increased by the strapping. This is usually due to turning a broken end inward. In any case the dressing is to be removed, and the patient must simply be kept as quiet as possible.

In some old men, chronic hemorrhoids are often due to straining in emptying the bladder, owing to enlarged prostate. In such cases it is better to advise the regular use of the catheter, even though the patient is able to thoroughly empty his bladder. The relief from straining commonly causes the piles to disappear.

When a patient first uses crutches, see that the arm pieces are thoroughly padded if any exclusive use of the crutches is expected. Paralysis, due to pressure on the nerves, is not uncommon. The musculospiral is most frequently involved, giving wrist-drop. The ulnar is also sometimes injured, but any or all of the nerves may be affected.

In the presence of any joint affection, the first object of treatment should be to obtain complete rest. The next one is to secure such position that, should ankylosis take place, the limb will be most advantageously placed. In inflammatory affections, especially in children, anæsthesia may be needed to obtain the position required.

There are certain regions, in which permanent cure after removal for cancer occurs rather rarely. It frequently happens, however, that death from recurrence is less painful than death from the primary affection. This is noticeably so in case of the tongue, and should be considered among the indications for operation.

In non-penetrating wounds of the abdomen, we may have considerable bleeding from the injury to the circumflex iliac, internal mammary and epigastric arteries. If the wound be penetrating, remember that the same cause of hemorrhage may be at fault. Such superficial hemorrhage should be looked for and checked, but no probing can be allowed. Non-penetrating wounds may, of course, be infected, and give rise to serious sepsis, with typhoid symptoms, etc. They are to be treated as anywhere else.—*Internat. Jour. Surgery.*

